# Therapy of traumatic brain injury by modern agents and traditional Chinese medicine

**DOI:** 10.1186/s13020-023-00731-x

**Published:** 2023-03-11

**Authors:** Chunzhu Wei, Jingbo Wang, Jintao Yu, Qing Tang, Xinjie Liu, Yanlong Zhang, Dandan Cui, Yanqiong Zhu, Yanli Mei, Yanjun Wang, Wenzhu Wang

**Affiliations:** 1 Department of Integrated Traditional and Western Medicine, Union Hospital, Tongji Medical College, Huazhong University of Science and Technology, Wuhan, China; 2 Department of Otolaryngology, Union Hospital, Tongji Medical College, Huazhong University of Science and Technology, Wuhan, China

**Keywords:** Traumatic brain injury, Pharmacological therapies, Traditional Chinese medicine, Modern agents

## Abstract

Traumatic brain injury (TBI) is the leading cause of disability and death, and the social burden of mortality and morbidity caused by TBI is significant. Under the influence of comprehensive factors, such as social environment, lifestyle, and employment type, the incidence of TBI continues to increase annually. Current pharmacotherapy of TBI mainly focuses on symptomatic supportive treatment, aiming to reduce intracranial pressure, ease pain, alleviate irritability, and fight infection. In this study, we summarized numerous studies covering the use of neuroprotective agents in different animal models and clinical trials after TBI. However, we found that no drug has been approved as specifically effective for the treatment of TBI. Effective therapeutic strategies for TBI remain an urgent need, and attention is turning toward traditional Chinese medicine. We analyzed the reasons why existing high-profile drugs had failed to show clinical benefits and offered our views on the research of traditional herbal medicine for treating TBI.

## Background

Traumatic brain injury (TBI) leads to extensive neurologic disability and mortality rates worldwide [1]. Diagnoses range from mild TBI to moderate and severe TBI depending on the severity of the injury. The incidence of TBI has increased during the last few decades, with an important impact on public health. It has been estimated that more than 60 million people worldwide remain injured after experiencing TBIs of varying severities [2]. Falls as well as motor vehicle and work-related accidents are the most frequent causes of TBI [3, 4]. Despite the progress that has been made in diagnosis, neurosurgical care, and functional rehabilitation, no effective therapy is currently available for TBI.

**Fig. 1:**
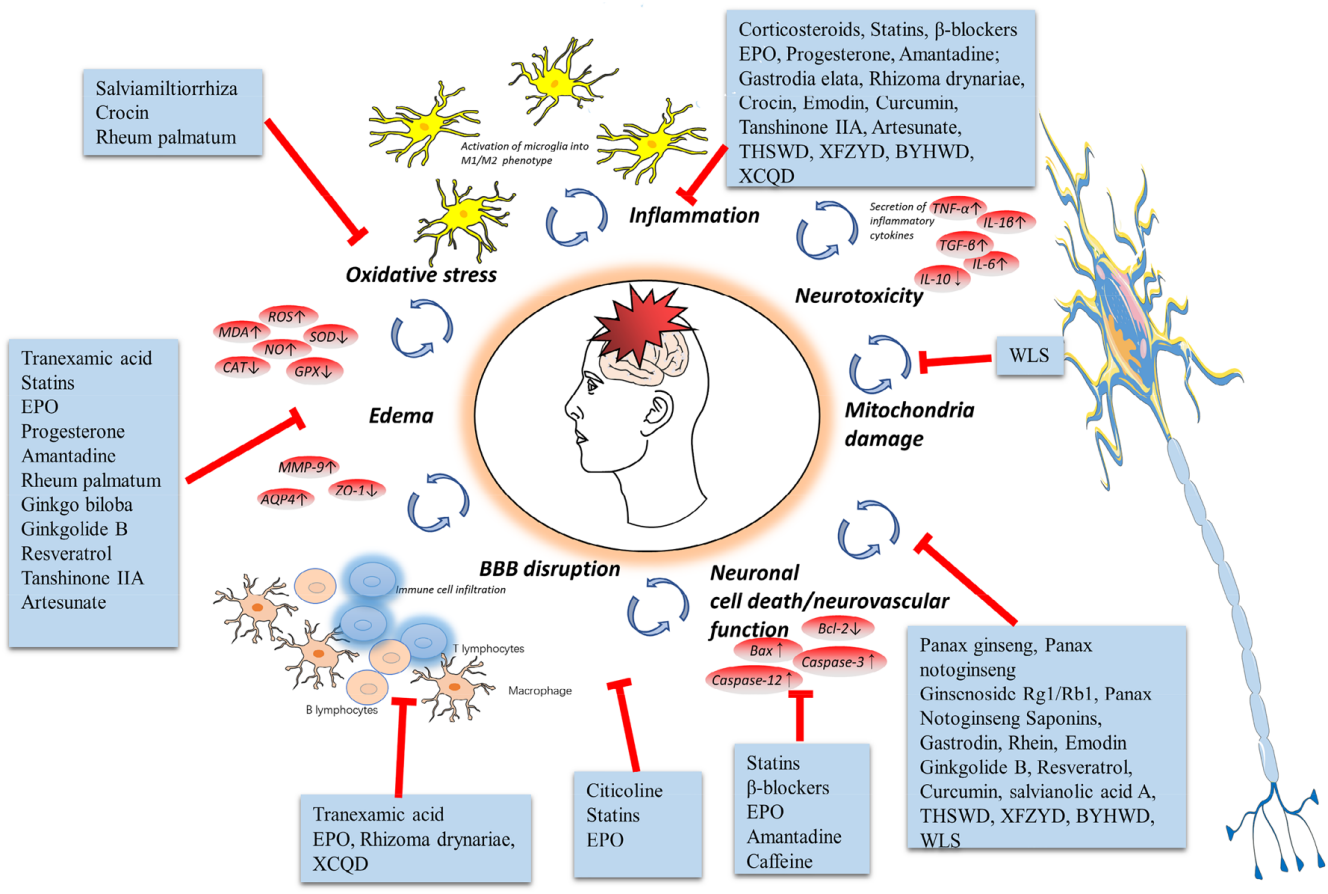
Multipotential drug treatment strategies for TBI

Multiple drugs have been studied to treat TBI in animal models and patients. Among them, hyperosmotic drugs were used for reducing intracranial pressure; anticoagulant drugs were used to treat deep venous thrombosis in the brain; antiepileptic drugs were used for preventing or controlling epilepsy; and antibiotics were applied to prevent or control infection (Fig. 1). Currently, many new drugs are being examined in clinical trials [5]; meanwhile, many types of existing drugs that have been used to treat other diseases are being trialed for TBI [6], with some even having entered phase III clinical trials (Table 1). In addition, an increasing number of researchers have tested herbal medicines and achieved good results [7]. This review sought to summarize and assess the effectiveness of these drugs for TBI (Tables 2, 3, 4, 5) and offered some expectations of research in the future.

**Table 1:** Clinical trials in patients with TBI per trial phase

Status	Study phases					Percentage of studies per status
	Early phase I	I	II	III	IV	
Completed	0.70%	7.10%	16.40%	11.10%	8.60%	43.90%
Withdrawn/Terminated	0.70%	1.40%	10.70%	6.10%	3.60%	22.50%
Recruiting	1.80%	2.10%	3.90%	3.20%	2.50%	13.50%
Not yet recruiting	1.10%	0	3.90%	2.10%	1.10%	8.20%
Unknown	1.10%	0.70%	5.40%	1.40%	3.20%	11.80%
Sum	5.40%	11.40%	40.30%	23.90%	19.00%	100%

**Table 2:** Treatment with modern drugs of entering clinical trials in animal models post-TBI

Multipotential modern treatment strategies	Models	Species	Sample size	Gender	Mode of action	Effects on post-TBI consequences
Corticosteroids [8-11]	CCI	Rat	36	Male	↓ the number of CD31^+^ cells and circulating EPCs	↓ neuronal cell loss and impaired neurofunction
	CCI	Rat	296	Male	↑ MR; ↓GR	↓apoptosis and cell loss, and impaired spatial memory
	CCI	Rat	Unknown	Male	↓ TGF-β1, IL-1β, TNF-α,↑ IL-10) ；↓ Bax, ↑Bcl-2	↓cavity size;↑neuronal cell survival and improved motor functional recovery
	CCI	Rat	24	Male	↓NeuN^+^ and apoptotic cells; ↓ activated microglia/infiltrated macrophages ; ↓ IL-1β, TGF-β1, TNF-α, and IFN-γ	↓lesion volume; ↑motor and cognitive function
Citicoline [23-25]	WDI	Rat	30	Male	↓S-100β and NES	↑nerval protection effect
	WDI	Rat	40	Male	↓S-100β and IL-6	↑brain functional recovery
	WDI	Rat	40	Male	↓neurological severity score, balance test;↑slanting board test	↑ the recovery of neural dysfunction
β-blockers [20, 21]	CCI	Mouse	64	Male	↓leukocyte rolling; ↑GNT	↓edema
	WDI	Mouse	100	Both sexes	↓p-tau	↓learning and memory deficit
Statins (atorvastatin, simvastatin, lovastatin) [28-30, 32]	WDI	Mouse	36	Male	↓TNF-α, IL-1β, TBK1,TLR4; ↓microglia activation	↓edema; ↓cognitive deficit
	WDI	Rat	18	Male	↓TNF-α, IL-1β, NF-κB, CD40	↓TBI-induced intestinal injury
	FPI	Rat	30	Male	↓ TLR4,NF-KB p65,p-IκB,cleaved Caspase-3, IL-6, TNF-α and IL-1β	↑neurological function
	FPI	Rat	32	Male	↓GRP78, P-PERK,P-eif2α, ATF6,Caspase12,CHOP, Nrf2 and Caspase3	↓edema; ↑neurological function
Tranexamic acid [39-42]	WDI	Mouse	60	Male	↓ MCP-1 , IL-6, TNF-α, p-tau and NSE; ↑ MIP-1α	↓ neuroinflammatory response
	CCI	Pig	20	Female	↓ p-tau and IL-1β	↑neurological function
	CCI	Mouse	Unknown	Male	↑immune activation at the 72 h, altere several myeloid and lymphoid cell populations	↑immune-modulatory properties
	CCI	Mouse	Unknown	Both sexes	↓ blood neutrophils in male mice 3 h post-TBI, ↑ monocyte subsets and dendritic cells	protective effects post-TBI but only in male mice
Erythropoietin [46-49]	WDI	Rat	15	Male	↑BDNF and SDF-1; ↓NSE	↑anti-inflammatory properties
	CCI	Rat	76	Unknown	↓NF-κB and GR; ↑GPX, SOD, GSH/GSSG and HIF-1α	↓apoptosis and cell loss
	WDI	Mouse	Unknown	Male	↓GFAP and S100; ↑MAPK, cAMP, CREB and NOR	↑memory response, hippocampal neuroprotection, and neurogenesis
	WDI	Rat	20	Male	↑AQP4, ↓GFAP	↓brain edema
Progesterone [13-17]	CCI	Rat	121	Both sexes	↑NOR, EAAT-3 and NaVβ3; ↓EPSCs,IPSCs, frequency and EPSC amplitude	↑motor and memory functional recovery
	CCI	Rat	36	Female	↓ICP;↑CPP	↓ cerebral edema; ↓learning and memory deficit
	WDI	Rat	48	Male	↓Bcl-2; ↑BAX	↑motor and cognitive function
	WDI	Rat	210	Female	↑SOCS-3, p-STAT-3, NFκB-P52, p-NFκB-P65 and p-IκBα	↓ brain edema
	WDI	Rat	Unknown	Female	↑NGF; ↓ IL6	↓ cerebral edema
Amantadine [54, 55]	CCI	Rat	60	Male	↓ the numbers of degenerating neurons	↓brain lesion; ↓learning and memory deficit
	WDI	Rat	Unknown	Male	↓ DNA fragmentation	↓ neuronal cell loss and impaired neurofunction
Caffeine [62-64]	FPI	Rat	180	Male	↓lethal apnea (an acute bolus of caffeine)	↑motor function
	WDI	Rat	56	Male	↑spine density in OFC, mPFC and NAc; ↓telomere length	↓motor and cognitive function
	WDI	Rat	64	Both sexes	↑Drd1, Drd2, Gfap, Dnmt3a and longer telomeres	↓anxiolytic-like behaviors

*CCI* controlled cortical impact, *WDI* weight drop injury, *FPI* fluid percussion injury, *EPCs* endothelial progenitor cells, *MR* mineralocorticoid receptor, *GR* glucocorticoid receptor, *GNT* garcia neurologic test, *p-tau* phosphorylated tau, *NF-κB* nuclear transcription factor-κB, *IκB *inhibitor of NF-κB, *NSE* neuron-specific enolase, *GRP78* glucose-regulated protein 78, *PERK* proteinkinaseR—likeERkinase, *eIF2α* eukaryotic tanslation initiation factor 2α, *ATF6* activating transcription factor, *Caspase 3* cysteme aspartate specific protease 3, *Caspase 12* cysteme aspartate specific protease 12, *CHOP* CAAT/enhancer binding protein homologous protein, *Nrf2* nuclear factor (erythroidderived 2)-like 2, *MCP-1* monocyte Chemoattractant Protein-1, *MIP-1α* macrophage inflammatory protein 1α, *SDF-1* stromal cell-derived factor 1, *GPx* glutathione peroxidase, *GSH* reduced glutathione, *GSSG* oxidized glutathione, *GR* glutathione reductase, *SOD* superoxide dismutase, *NOR *novel object recognition, *MAPK* mitogen-activated protein kinase, *cAMP* cyclic adenosine monophosphate, *AQP4* aquaporin 4, *GFAP* glial fibrillary acidic protein, *EPSCs* excitatory post-synaptic currents, *IPSCs* inhibitory post-synaptic currents, *EAAT3* excitatory amino acid transporter 3, *NaVβ3* β subunit of the voltage-gated sodium channel, *ICP* Intracranial pressure, *CPP* cerebral perfusion pressure, *SOCS-3* suppressor of cytokine signaling-3, *STAT-3* signal transducer and activator of transcription-3

**Table 3:** The neuroprotective effects of herbal medicines on animal models post-TBI

Traditional Cinese medicines	Models	Species	Gender	Sample size	Mode of action	Effects on post‐TBI consequences
Panax ginseng [65, 66]	CCI	Mouse	Male	Unknown	↓ p-AMPKα, PGC-1α, ERRα, cytochrome c and MTCO2, O2 and ATP; ↑HO-1	↑ neurovascular function
	CCI	Mouse	Male	Unknown	↑HO-1, Nrf2, Tom20 and PGC-1α; ↑Nampt, SIRT1, SIRT2, and SIRT3	↑ activity of astrocytes and neural stem cells
Panax notoginseng [70, 71]	WDI	Rat	Male	36	↓ p62,Beclin1, autophagy, light chain-3 protein	↓ apoptosis cells
	WDI	Rat	Male	48	↓ p-p65, t-PA, PT, APTT and ET; ↑ t-PA/PAI-1, CD61, CD62	↓ intracranial hemorrhage
Gastrodia elata [74]	CCI	Rat	Female	Unknown	↓ astrocytes, TNF-α and IL-6	↓ inflammation
Rheum palmatum [77]	CCI	Rat	Male	80	↓ MMP-9, ROS and p-ERK; ↑ ZO-1	↓ brain edema
Ginkgo biloba [81]	WDI	Rat	Male	80	↓ MMP-2 and MMP-9	↓ brain edema
Curcuma longa/Polygonum cuspidatum/Salvia miltiorrhiza	–	–	–	–	–	–
Rhizoma drynariae [99, 100]	WDI	Rat	Male	72	↓ CD8 T cells, no affecting the expressions of IL-2 and CD4 cells	↑ immune-promoting, anti- inflammatory
	CCI	Rat	Male	88	↓ IL-6,↑IL-10, CD3 and CD4 T lymphocytes, blood monocyte	↓ lesion volume, regulate cognitive and behavior function

*p-AMPKα* phospho-AMP-activated protein kinase, *PGC-1α* peroxisome proliferator-activated receptor γ-coactivator-1α, *ERRα* estrogen-related receptor α, *MTCO2* cytochrome c oxidase subunit 2, *HO-1* heme oxygenase-1, *PGC-1α* peroxisome-proliferator-activating receptor-γ coactivator-1α, *Tom20* translocase of the outer membrane of mitochondria 20, *Nampt* nicotinamide phosphoribosyl transferase, *SIRT1/2/3* Sirtuin1/2/3, *APTT* activated partial thromboplastin time, *t-PA* thromboplastin time, *PT* prothrombin time, *MMP-9* Matrix metalloproteinase 9

**Table 4:** The neuroprotective effects compounds of Traditional Chinese medicines on animal models post-TBI

Compounds of Traditional Chinese medicines	Subordinate herb	Models	Species	Gender	Sample size	Mode of action	Effects on post‐TBI consequences
Ginsenoside Rg1 [67]	P. Ginseng	WDI	Rat	Male	48	↓ Exos-miR-21, MMP1/3/9, ↑TIMP3, VEGF, GFAP and p-P65/P65, ZO-1,occludin and claudin-5	↓ cerebrovascular endothelial injury
Ginsenoside Rb1 [68]		CCI	Rat	Male	36	↑ p-ERK1/2; ↓connexin40	↓ brain water content
Crocin [69]	C. tinctorius	CCI	Mouse	Male	150	↑ GSH, ↓ IFN-γ, TNF-α, MPO and MDA	↑anti-inflammatory and anti-oxidative effects; ↑ cognitive function
Panax Notoginseng Saponins [72]	Panax notoginseng	WDI	Rat	Male	45	↑ PI3K/Akt/mTOR, Bcl-2; ↓ Bax	↓ apoptosis cells
Gastrodin [75]	Gastrodia elata	WDI	Rat	Male	30	↓GSDMD, NLRP3, ASC, caspase-1,caspase-11 and IL-18	↓ pyroptosis
Rhein [79]	Rheum palmatum	WDI	Mouse	Male	44	↓ LDH, GSDMD, NLRP3, TLR4, MyD88, IL-1β, IL-18 and IFN-γ; ↑p10, p20 and p45	↑ cognitive function; ↓ pyroptosis
Emodin [80]		WDI	Rat	Male	50	↓ NF-κB p65, ICAM-1, S100B, IL-6 and TNF-α; ↑ Nrf2	↑ cognitive function
Ginkgolide B [82]	Ginkgo biloba	WDI	Rat	Unknown	30	↓ Omi/Htr A2, procaspase-3/9, cleaved PARP, XIAP	↓ apoptosis cells;↓ brain edema
Resveratrol [83–85]	P.cuspidatum	WDI	Mouse	Male	72	↓P-p38 MAPK; ↑ Sirtuin 1 and SYN	↑neurological function
		WDI	Mouse	Male	42	↑PGC-1, Sirtuin1and SYN	↑ cognitive function
		CCI	Rat	Male	150	↑P62,P-pI3K, P-Akt and P-mTOR; ↓LC3 II and Beclin-1	↓ brain edema; ↑ cognitive function
Curcumin [87-90]	C. longa	WDI	Rat	Male	36	↑ GFAP, DCX, Map2; ↓CD68	↓ brain lesion, apoptosis cells
		WDI	Mouse	Male	30	↓ROS, CXCL1, TNF-α, IL-1β, IL-6; ↑GAP43, doublecortin	↑cognitive function
		WDI	Mouse	Male	68	↓p-p38, NF-κB, IL-1β, IL-6 and TNF-α; ↑IL-10	↑neurological function; ↓inflammatory response
		CCI	Rat	Male	145	↑ BNDF, Trkb, PI3K and AKT;↑BrdU	↑ neurogenesis, spatial memory; ↓ inflammation
Salvianolic acid A [94]	S. miltiorrhiza	CCI	Mouse	Male	20	↓ TNF-α,IL-6,IFN-b,TLR2 and TLR4, NMDAR, PINK1,↑NMNAT1	↓ apoptosis
Tanshinone IIA [95]		WDI	Rat	Male	72	↓ p47phox, CD11, AQP4, GFAP, MDA, CAT, IL-1β, and TNF-α; ↑SOD and GSH-PX	↓brain edema,inflammation
Artesunate [96, 97]	A. annua	CCI	Mouse	Male	40	↓ Nf-kb, inflammation body complex, GFAP and Iba-1; ↑BDNF, GDNF and NT-3	↓inflammation
		CCI	Rat	Male	30	↓ GSK-3β; ↑occludin, ZO-1 Phosphorylation of Akt	↓ brain water content and neuroinflammation

*TIMP3* tissue Inhibitors Of metalloproteinase 3, *PI3K* Phosphoinositide 3-kinase, *GSDMD* Gasdermin D, *ASC* apoptosis-related macular protein of the adapter protein containing CARD, *NLRP3* NOD-like receptor protein 3, *ICAM-1*intercellular cell adhesion molecule-1, *VEGF* vascular endothelial growth factor, *GFAP* glial fibrillary acidic protein, *XIAP* X-linked inhibitor of apoptosis protein, *LC-3II* microtubule-associated-proteinlight-chain-3II, *SYN* synaptophysin, *Trkb* tropomyosin receptor kinase B

**Table 5:** The neuroprotective effects of Formulae and Decoctions of Traditional medicines on animal models post-TBI

Formulae and Decoctions	Models	Species	Gender	Sample size	Mode of action	Effects on post‐ TBI consequences
Taohong-Siwu Decoction [102]	WDI	Rat	Male	126	↑ Wnt, β-catenin, BDNF and NGF	↑ nerve regeneration
Xuefu-Zhuyu Decoction [104, 105]	CCI	Rat	Male	60	↓ Ppfia3, Rab11a, Rnf216 and Rnf6	↑ spatial memory
	CCI	Rat	Male	36	↑ metabolic profiles	↑ neurological function
Buyang-Huanwu Decoction [102, 107]	WDI	Rat	Male	306	↑ Wnt, β-catenin, BDNF and NGF	↑ nerve regeneration
	WDI	Rat	Male	126	↓ GFAP and Tau	↓ the risk of death
Wuling San [108]	WDI	Mouse	Male	56	↑ PGC-1α and NRF1; ↑mitochondrial biosynthesis	↓ brain water content;↑ Supplement of energy to the brain tissue
Wendan decoction	-	-	-	-	-	-
Da Chengqi Decoction [112]	WDI	Mouse	Male	150	↓ DAO and D-lactic acid; ↑occludin	↓gastrointestinal impairment
Xiaochengqi Decoction [113, 114]	WDI	Rat	Male	108	↓AQP4, TNF-α and IL-1β	↓ brain edema
	WDI	Rat	Male	90	↓ IL-1β, Bacteroidetes, Proteobacteria, Elusimicrobia, actinobacteria; ↑ Firmicutes and Spirochetes	↓harmful bacteria; ↓ inflammatory response

*BDNF*, brain-derived neurotrophic factor, *NGF* nerve growth factor, *GFAP* glial fibrillary acidic protein, *PGC-1α* peroxlsome proliferator-activated receptor-γ coactlvator-1α, *NRF1* nuclear respiratory factor 1, *DAO* diamine oxidase

## Modern agents

### Inclusion criteria

PubMed [National Library of Medicine/National Institutes of Health (NLM/NIH)], Web of Science [Institute of Scientific Information, ISI], and ClinicalTrials.gov [National Library of Medicine/National Institutes of Health (NLM/NIH)] were searched for privately and publicly funded clinical studies conducted around the world. All studies were conducted before 23 January, 2023. The databases were searched using both controlled vocabulary words and synonymous free-text words for the topic of interest (TBI, agent, drug, medicine, and medication). This review included clinical trials, randomized controlled trials (RCTs), controlled clinical trials, and observational studies; both sexes (female and male); all age stages [child (birth–17 years), adult (17–65 years), and older adult (≧65 years)]; and all study phases. Approximately 120 drugs have been or will be investigated in 512 clinical trials. We focused on therapies approved by the United States Food and Drug Administration (FDA) or European Medicine Agency (EMA), which were widely applied in indications for other diseases, as they should have a decent chance of succeeding. Considering their well-known side effects, it is easier to assess the benefit/risk stability of these drugs. Therefore, we restricted “modern agents” to the following potential strategies that have been extensively examined in animal models of TBI: corticosteroids, progesterone, β-blockers, citicoline, statins, tranexamic acid, erythropoietin (EPO), amantadine, caffeine.

### Corticosteroids

Corticosteroids have been widely used to treat inflammatory-mediated and immune-related diseases because of their anti-inflammatory and immunosuppressive effects. It is well known that secondary brain edema and swelling increase intracranial pressure, leading to further distortion of the structure of the brain as well as reduced cerebral blood perfusion and oxygen content. To date, corticosteroids have been used to treat TBI for more than 40 years. Previous animal studies of TBI have shown that treatment with corticosteroids exerts neuroprotective effects by reducing the expression of CD31^+^ cells and circulating endothelial progenitor cells (EPCs) [8], glucocorticoid receptor (GR) [9], TGF-β1, IL-1β, TNF-α [10], and activated microglia/infiltrated macrophages [11], as well as by increasing the expression of mineralocorticoid receptor (MR) and IL-10.

A large-scale multicenter double-blinded International Standard Randomized Controlled Trial (ISRCTN74459797), Corticosteroid Randomization After Significant Head Injury (CRASH), randomized 10,008 patients with TBI to a 48-h infusion of 0.4 mg corticosteroid versus placebo [12] and explored the risk of death and disability after TBI. The investigators restricted study participation to patients who were at least 16 years old and had a Glasgow Coma Scale (GCS) score of no more than 14 points in the hospital, while those with contraindications to glucocorticoids were excluded from the study. The primary end point was the rate of deaths within 14 days after injury or the presence of disability up to 6 months later. The results indicated that corticosteroid treatment significantly increased the 2-week mortality rate compared with placebo (17.9% vs. 21.1%; P < 0.001), but there was no difference between the two treatment arms regarding disability at 6 months. Moreover, there was no difference in the composite outcome of certain complications, such as hypertension and immunosuppression. Based on this study, corticosteroids are not recommended for routine use in the treatment of TBI.

### Progesterone

Progesterone is a female steroid hormone that easily crosses the blood–brain barrier and is well tolerated. Notably, progesterone has potent neuroactive and neurosteroid effects in the treatment of brain injury. Many preclinical studies have reported that progesterone improves motor and cognitive function by reducing the levels of excitatory postsynaptic currents (EPSCs), inhibitory postsynaptic currents (IPSCs), intracranial pressure (ICP), Bcl-2, and IL-6, as well as by increasing the expression of excitatory amino acid transporter 3 (EAAT-3), β subunit of the voltage-gated sodium channel NaVβ3, suppressor of cytokine signaling-3 (SOCS-3), phosphorylated signal transducer and activator of transcription-3 (p-STAT-3), NF-κB-P52, p-NF-κB-P65, and nerve growth factor (NGF) in experimental TBI models [13–17]. Meanwhile, in two large double-blinded phase III RCTs (ProTECT III and SyNAPSe), more than 2,000 patients were investigated. Those studies failed to demonstrate a clinical benefit in terms of postoperative mortality and functional outcomes after TBI [18, 19], even resulting in the termination of subsequent clinical trials investigating progesterone.

### β-Blockers

β-Blockers, a class of cardio-cerebrovascular–protective drugs, play a major role in preventing the release of excitatory neurotransmitters, thereby promoting nerve injury repair in TBI. In recent years, a growing amount of experimental evidence collected from animal studies has suggested that β-blockers have neuroprotective effects against TBI via a mechanism that involves the reduction of leukocyte rolling and p-tau [20, 21].

Schroeppel et al. [22] reported that propranolol had a protective effect on TBI and reduced the mortality rate in a prospective randomized pilot trial, despite their failure to show differences between the exposed and nonexposed arms. Similar results were found in a prospective RCT [6], where 119 patients (age ≥ 18 years) were randomly divided into a group that was given 20 mg propranolol orally every 12 h for up to 10 days or until discharge from the hospital and a group that did not receive propranolol. No significant difference in the in-hospital mortality rate was recorded between patients exposed to β-blockers and those not exposed. In contrast, patients who received propranolol experienced dramatically decreased mortality compared with those in the unexposed arm after applying specified exclusion criteria, which satisfied the conditions that “severe TBI = intracranial abbreviated injury scale (AIS) ≥ 3, with all extracranial injuries ≤ 2.” Despite its prospective randomized design, the current 14 trials were limited by many confounding factors. First, some trials lacked blinding or placebo and were limited by the involvement of one trauma center only. Second, more homogeneous research population and multicenter studies were lacking. Mortality rates among TBI patients with different conditions were not comparable. Finally, the control of single-dose β-blockers was not suitable during hospitalization, causing some patients not to receive the same number of single-dose β-blockers during the trial.

### Citicoline

Citicoline is an activator of brain metabolism that can develop into cytidine (or uridine in humans) and choline through hydrolysis and dephosphorylation. Subsequently, citicoline is resynthesized by hydrolysis and dephosphorylation as the material base of cytidine and choline after passing the blood–brain barrier. As a result, it plays an intracellular neuroprotective role by supporting the biosynthesis of phospholipids. Therefore, citicoline is considered a potential drug for the treatment of partial secondary brain injury. Currently, many preclinical studies have focused on the neuroprotective effects of cytidine on TBI. The mechanisms involve anti-inflammation by the reduction of S-100β, IL-6, and neuron-specific enolase (NSE) [23–25]; However, the neuroprotective effect of citicoline remains unproven in clinical trials.

The only citicoline clinical phase III trial in TBI, a double-blinded randomized trial known as Citicoline Brain Injury Treatment (COBRIT) trial that was performed in the United States from July 20, 2007, to February 4, 2011, investigated whether citicoline administered within 24 h could improve functional and cognitive status in humans with mild, moderate, or severe TBI. A total of 607 patients were randomized to receive citicoline at a dose of 1,000 mg twice daily for 90 days by an oral or enteral administration route, and 606 patients were administered a placebo. This study revealed that acute and subacute treatment with citicoline failed to yield evidence of amelioration in brain function and cognition relative to placebo. However, a subsequent secondary analysis using recovery curves with repeated measures showed that most patients had favorable outcomes at 180 days after injury in all TBI groups. There was substantial amelioration in all of the groups from 1 to 180 days after injury, and this improvement can last beyond 180 days [26].

More studies have adopted similar approaches and applied multimodal and longitudinal datasets to analyze their results [27]. However, additional studies are needed to prove the therapeutic effect of citicoline, and currently, there is insufficient evidence available to support the recommendation of citicoline in patients with TBI.

### Statins

Statins, also known as 3-hydroxy-3-methyl glutaryl coenzyme A (HMG-CoA) reductase inhibitors, are a kind of medication generally prescribed to treat elevated cholesterol levels. Statins work to competitively inhibit HMG-CoA reductase by blocking the intracellular hydroxyvalerate metabolism pathway and reducing endogenous cholesterol synthesis, which leads to reflexive stimulation of the quantity and activity of low-density lipoprotein receptors on the surfaces of cell membranes, thereby reducing the level of cholesterol in serum [28]. Previous studies [28–30] have confirmed the beneficial effects of these drugs not only in hyperlipidemia but also in TBI. Moreover, previous animal studies [28, 29] of TBI have shown that treatment with statins exerts neuroprotective effects by increasing the expression of endothelial nitric oxide synthase (NOS), promoting neurogenesis and synaptogenesis, increasing angiogenesis, and enhancing antioxidant and anti-inflammatory properties. Possible mechanisms are associated with anti-inflammation and anti-apoptosis effects of nerve cells by the reduction of microglia activation, TNF-α, IL-1β, NF-κB, CD40, TANK binding kinase 1 (TBK1), Toll-like receptor 4 (TLR4), glucose-regulated protein 78 (GRP78), and Nrf2 [28–32]. However, few studies have focused on the effects of statins on the functional prognosis of humans with TBI.

A retrospective study investigated differences in in-hospital mortality rates between prior statin users and nonusers among TBI patients. A total of 116,193 patients were included in this study, and the statins in question included atorvastatin, lovastatin, fluvastatin, rosuvastatin, pravastatin, and simvastatin. The authors concluded that statins can significantly reduce in-hospital mortality after TBI [30]. Moreover, pre-TBI statin use could be related to a survival benefit and improvements in neurologic function in TBI patients [33]. However, it should be noted that these findings were gathered from a single-center observational study of the older patient population. Furthermore, a phase II clinical trial explored whether the administration of atorvastatin for 7 days after injury was safe in 140 patients with mild TBI randomized to receive either atorvastatin (1 mg/kg per day for 7 days) or placebo within 24 h of injury. No serious adverse effects associated with atorvastatin treatment occurred, and the authors concluded that the administration of atorvastatin for 7 days after TBI was safe but not effective in the improvement of functional outcomes, such as neurologic recovery [34]. Similar conclusions have been reached in the recent studies conducted by Soltani et al. [35] and Mansi et al. [36]. Although their results seemed to suggest some progress, they were limited by using a retrospective study design, small sample size, or single severity degree of TBI, and larger prospective RCTs are required to evaluate the potential effects and clinical benefits of statins after TBI.

### Tranexamic acid

Tranexamic acid (TXA) is a derivative of lysine. It is known as an anti-fibrinolytic drug, which does not increase the synthesis of fibrin in the process of hemostasis. It exerts hemostatic effects by reversing binding to the lysine site to block binding on plasminogen, thereby reducing the level of fibrinolysis [37]. Hemorrhage and edema are associated with poor clinical outcomes in TBI. From a theoretical view, TXA can achieve hemostasis without increasing the risk of thromboembolic events [38] and is a potential agent for the treatment of TBI.

In the past decade, the potential effects of TXA in animal models of TBI have been confirmed. Studies of TBI have shown that treatment with TXA exerts neuroprotective effects of immunoregulation and anti-inflammation by increasing the levels of macrophage inflammatory protein 1α (MIP-1α), lymphoid cell populations, monocyte subsets, and dendritic cells, and reducing the levels of monocyte chemoattractant protein-1 (MCP-1), IL-6, IL-1β, TNF-α, p-tau, and NSE [39–42]. Clinical trials have also drawn increasing attention. We summarize three randomized, placebo-controlled trials in which the potential brain-protective effects of TXA therapy after TBI were investigated in the setting of bleeding trauma. In one case–control study (NCT00375258), Clinical Randomization of an Anti-fibrinolytic in Significant Hemorrhage 2 (CRASH-2), the effects of TXA therapy on death, vascular occlusive events, and blood transfusion in 20,211 patients with bleeding trauma were investigated at 274 hospitals in 40 countries. The results suggested that TXA dramatically reduced mortality in terms of the absence of increased vascular occlusive events but failed to confirm less mortality in a TBI subgroup [43]. Moreover, researchers further evaluated the effects of TXA in patients with TBI in CRASH-3 (NCT01402882), where 12,737 patients with TBI were randomly allocated to a TXA group, where they received a 1-g loading dose over 10 min, followed by an infusion of 1 g over 8 h, or a matching placebo group. This study offered evidence that TXA is safe in patients with TBI and can reduce TBI-related deaths. Importantly, the optimal time window of treatment is within 3 h of injury [44].

However, CRASH-3 only included patients with mild TBI who showed intracranial hemorrhage on computed tomography scans, and it remains to be determined whether its results could be applied to patients with mild TBI more generally. Thus, the foundation for CRASH-4 (NCT04521881)—which plans to seek reliable evidence of the effects of early intramuscular TXA on disability, death, and dementia in older patients with intracranial hemorrhage with symptomatic mild TBI—has been laid. Furthermore, another forthcoming study (NCT04387305) designed to assess the benefits and harms of TXA in severely traumatic children with hemorrhagic brain injuries and/or injury to other parts, such as the trunk, is eagerly anticipated.

### Erythropoietin (EPO)

EPO is a glycoprotein produced by the kidneys that regulates bone marrow hematopoiesis, resulting in the natural production of erythrocytes following hypoxic stimulation. EPO production occurs not only in the bone marrow, liver, spleen, and lungs but also in the brain. Currently, the two main indications for EPO agents approved by the FDA are anemia secondary to chronic kidney disease in patients with nephrosis and chemotherapy in cancer patients [45]. Preclinical studies have shown that EPO acts as a neurotrophic factor, which can relieve the impact of secondary brain damage by the blockade of the apoptotic program, improvement of neurogenesis, and inhibition of an inflammatory response. The specific mechanisms are associated with the reduction of NF-κB, NSE, GR, GFAP, and S100 and the elevation of GPX, SOD, GSH/GSSG, HIF-1α, mitogen-activated protein kinase (MAPK), cyclic adenosine monophosphate (cAMP), and aquaporin 4 (AQP4) [46–49]. However, it remains controversial whether EPO has neuroprotective effects in TBI.

In a double-blinded RCT with 159 patients, the short-term efficacy of early treatment with recombinant human EPO after severe TBI was investigated. Treatment was initiated within the first day in patients with a GCS score of 8 points or less upon arrival, who received EPO (100 units/kg) or placebo on days 1, 3, 6, 9, and 12 after injury. It was demonstrated that patients in the EPO treatment group had lower GCS scores and lower levels of NSE and S-100β protein in serum than the placebo group following the first 3 months after administration. The authors concluded that recombinant human EPO can improve three-month functional recovery in patients with severe TBI and that it does not increase the incidence of thromboembolic events or severe infection [50]. However, in a large multicenter, double-blinded, parallel-group RCT (NCT00987454) with 606 patients with moderate to severe TBI, Skrifvars et al. [51] evaluated the efficacy of EPO treatment (40,000 IU subcutaneously per week) versus placebo (0.9% physiological saline) and found that EPO administration did not improve severe neurologic dysfunction or reduce 6-month mortality. Similar findings were confirmed in other studies, without an increased risk of deep vein thrombosis events or severe infections [50, 52, 53]. However, these studies had several limitations, including the small sample size and the absence of long-term outcome follow-up data. Therefore, a new study, Erythropoietin Alfa to Prevent Mortality and Reduce Severe Disability in Critically Ill Trauma Patients (NCT04588311), is currently recruiting participants.

### Amantadine

Amantadine is an indirectly acting dopamine agonist with presynaptic and postsynaptic effects. In addition, it is a noncompetitive N-methyl-d-aspartate antagonist. The neuroprotective effect of amantadine in experimental TBI has been confirmed. Previous studies have found that the administration of amantadine could improve neural regeneration in rats with TBI by reducing the expression of degenerating neurons and DNA fragmentation [54, 55].

In a small, double-blinded, single-center, phase II RCT study, 42 patients with severe TBI were randomized to receive either amantadine or a placebo for 6 weeks. GCS and FOUR scores were recorded on days 1, 3, and 7 following treatment with placebo or amantadine, and Mini-Mental State Examination, Glasgow Outcome Study, Disability Rating Scale scoring, and Karnofsky Performance Scale scoring were conducted after the drug was administered. The authors concluded that there was essentially no effect on the behaviors, consciousness, cognitive function, and mortality of patients with severe TBI at 6 months [56]. Another multicenter, parallel-group, double-blinded RCT investigated the effects of amantadine on cognition in 119 patients with chronic TBI (lasting 6 months or more postinjury) and did not find significant effects of amantadine treatment, even showing that cognitive processing may be hampered during the first 28 days of administration of amantadine [57].

However, a recent triple-blinded, placebo-controlled randomized study evaluating the effects of amantadine on brain dysfunction in 57 patients demonstrated that amantadine can hasten the recovery of functional ability in patients with severe TBI [58]. Unfortunately, the study did not have a large sample size and offered long-term data only for a single population (i.e., for patients from the Imam Reza Hospital neurosurgery ward). Some researchers have found that the combination of amantadine and other drugs, such as citicoline, Cerebrolysin, and memantine, may improve prolonged disorders of consciousness in patients with TBI [59, 60].

Two relevant phase IV clinical studies, Combination Treatment with Cerebrolysin and Amantadine on Patients Who Stay in Prolonged Disorders of Consciousness Due to Severe Traumatic Brain Injury and the Citicholine–Amantadine Trial in Traumatic Brain Injury (NCT04427241), are currently in progress.

### Caffeine

Caffeine is one of the most widely consumed addictive compounds by a huge population worldwide and has multiple effects on the central nervous system. It is well known that extreme doses of caffeine can cause neurotoxicity [61]. Notably, common-dose of caffeine can be a double-edged sword in TBI models of different genders and/or therapeutic windows.

Lusardi et al. [62] demonstrated that instead of caffeine withdrawal, chronic caffeine therapies (0.3 g/L caffeine exposure for 3 weeks pre-TBI; 0.3 g/L caffeine exposure for 1 week post-TBI) impeded the improvement of motor function. More importantly, despite chronic caffeine preexposure, a single acute dose of caffeine (25 mg/kg) after TBI can prevent lethal apnea and has no negative impacts on motor function after sublethal injuries. A subsequent study showed that a common dosage of caffeine (1 g/L, approximately equivalent to five cups of coffee per day) caused the greatest detriments to both behavioral and cognitive functions in males, while the most considerable impairments in females were related to tasks associated with pain. Caffeine elevated spine density in the orbitofrontal cortex (OFC), medial prefrontal cortex (mPFC), and nucleus accumbens (NAc), which was thought to be due to damage in the routine pruning processes following TBI [63]. In a rather unusual study, before mating with control females, sires were kept in conventional cages or cages with running wheels and were given standard drinking water, caffeine, or ethanol for 7 weeks. At postnatal day 40, infants received TBI or sham injuries, and their postconcussive symptoms were evaluated. This study showed that offspring of caffeine-treated sires had higher levels of dopamine receptor D1 (Drd1), dopamine receptor D2 (Drd2) Drd2, GFAP, and DNA methyltransferase 3A (Dnmt3a) expression, as well as longer telomeres, which offered additional proofs that paternal caffeine exposure before conception affects the development of the offspring [64]. More preclinical and clinical research should be done in the future because existing studies on caffeine's effects in the treatment of TBI have shown results that appear to be contradictory.

### Traditional herbal medicine

Modern medicine is largely dominated by phases I, II, III, and even IV clinical trials (Table 1); meanwhile, herbal medicines are widely used all over the world. Herbal medicines have been tested on humans for thousands of years instead of being tested on animals, and concrete effects have been recorded. Among all of the drugs approved by the FDA, 25% are derived from traditional herbal medicines [7]. We highlight the potential neuroprotective value of traditional Chinese herbal medicine in TBI, including combination formulas, single herbs, and active compounds, as well as their mechanisms of action.

### Single herbs and their active compounds

*Panax ginseng (P. ginseng)*, also known as ren shen, is native to China and Korea and is sourced as the dried root of a plant in the Araliaceae family [65]. The actions of *P. ginseng* were investigated post-TBI in a controlled cortical impact (CCI) mouse model, and the possible mechanisms were related to the MAPK/NF-κB signaling pathway [66] and HO-1-mediated AMPKα-PGC-1α-ERRα circuit after TBI [65]. *P. ginseng* contains many active compounds, such as *Rg1*, *Rb1*, and *Rb2*. Treatment with *ginsenoside Rg1* in a weight drop injury (WDI)-induced TBI rat model ameliorated cerebrovascular cellular damage by elevating the expression levels of TIMP3, VEGF, GFAP p-P65/P65, ZO-1, occluding, and claudin-5, and by hampering the levels of exos-miR-21 and MMP1/3/9 [67]. Zou et al. [68] found that *ginsenoside Rb1* treatment exerted a neuroprotective effect in rats with TBI via reversing the fluid percussion injury (FPI)-induced downregulation of connexin40, and enhancing the expression levels of p-ERK1/2.

*Carthamus tinctorius* L. is a dried flower of the composite family that has been used in traditional herbal medicine to treat various conditions, including thrombosis, menstrual flow irregularities, spasms, and flatulence. *C. tinctorius* L. contains many active compounds, such as *crocin*, *crocetin*, *safranal*, and *isophorone*, although so far researchers have only found that crocin has a neuroprotective role in TBI. Treatment with crocin in a CCI-induced TBI mouse model enhanced the anti-inflammatory and antioxidant effects by increasing the expression levels of NSS and GSH and by reducing the expression levels of IFN-γ, TNF-α, MPO and MDA [69].

*Panax notoginseng*, also known as sanqi, belongs to the Araliaceae family. *P. notoginseng* is known as “the miraculous agent capable of stopping the blood” and is commonly used for promoting blood circulation, relieving blood stasis, and alleviating pain in China. *P. notoginseng* contains many active compounds, such as *P. notoginseng saponins* (PNS), dencichine, flavones, and ginsenoside [70]. Thus far, researchers have found that ginsenoside has a neuroprotective role in TBI. Jiang et al. [71] found that the administration of *P. notoginseng* had neuroprotective effects against TBI by reducing the level of p-p65, thromboplastin time (t-PA), prothrombin time (PT), activated partial thromboplastin time (APTT), and endothelin (ET), and by elevating the levels of CD61 and CD62. Furthermore, an injection of *PNS* intraperitoneally after the induction of TBI in a WDI rat model improved behavioral test results via increasing the expression of phosphatidylinositol 3-kinase (P13K), Akt, and mTOR [72].

*Gastrodia elata* belongs to the Orchidaceae family. Its dried rhizome is commonly used for the treatment of neurologic disorders, such as headache, stroke, Alzheimer's disease, and vertigo [73]. In a rat model of TBI, the administration of *G. elata* aqueous extract showed neuroprotective effects against TBI. It also improved the rotarod test results and reduced the number of astrocytes, TNF, and IL-6 [74]. *G. elata* contains many active compounds, such as gastrodin, 4-hydroxybenzaldehyde, and vanillin; among them, gastrodin has been confirmed to have neuroprotective effects against TBI. The administration of *gastrodin* after the induction of TBI by CCI in a rat model remarkably suppressed pyroptosis and affected the anti-inflammatory action of nerve cells of rats by inhibiting the expression of Gasdermin D (GSDMD), NLRP3, ASC, caspase-1, and caspase-11 and by reducing proinflammatory cytokines such as TNF-α, IL-1β, and IL-18 [75]. Of note, in China, *gastrodin tablets*, a nonprescription drug extracted from *G. elata*, have been approved for use in neurasthenia, headache, and migraine by the State Drug Administration [76].

*Rheum palmatum* is sourced as the dried root of a plant in the Polygonaceae family. The effect of *R. palmatum* after TBI was investigated in a rat model created by CCI. It was shown that its aqueous extract was able to relieve edema by impeding the extracellular signal–regulated kinase signaling pathway, reducing the levels of matrix metalloproteinase 9 (MMP-9), ROS, and p-ERK, and increasing the level of ZO-1 [77]. Moreover, *anthraquinone glycosides* from *Rhubarb* can play a protective role in rats with cerebral injury by increasing the levels of 5-hydroxytryptamine (5-HT), 5-hydroxy indole acetic acid (5-HIAA), and γ-aminobutyric acid (GABA), and contents of glutamic acid (Glu) and aspartic acid (Asp) [78]. *Rhein* and *Emodin* are isolated from *R. palmatum* and have neural benefits in TBI. *Rhein* can be absorbed by brain tissues following oral administration in TBI rats, which prevents neurologic impairment in mice following TBI by inhibiting neuronal pyroptosis. Moreover, *Rhein* remarkably decreases the levels of lactate dehydrogenase (LDH), GSDMD, NOD-like receptor protein 3 (NLRP3), TLR4, MyD88, NLRP3, IL-1β, IL-18, and IFN-γ, and increases the content of p10, p20, and p45 [79]. In vivo, *emodin* can exert neuroprotective effects by elevating NF-κB p65, intercellular cell adhesion molecule-1 (ICAM-1), S100B, IL-6, and TNF-α, and by suppressing NF-κB p65, ICAM-1, S100B, IL-6, and TNF-α [80].

*Ginkgo biloba* is collected in the form of a dried leaf from a plant of the Ginkgoaceae family. The effect of *G. biloba* extract was evaluated in a post-TBI rat model caused by CCI. The oral administration of *G. biloba* extract after injury in rats remarkably improved NSS and reduced brain edema. *G. biloba* also diminished the expression levels of VEGF, phosphatidylinositol3-kinase (PI3K), p-AKT, and MMP-9 to reduce oxidative stress and inflammation [81]. *Ginkgolide B* is isolated from *G. biloba* and has a neural benefit in TBI. The oral administration of *ginkgolide B* following the induction of TBI by WDI in a rat model remarkably reduces the number of apoptotic nerve cells by a reduction in Omi/Htr A2, procaspase-3, procaspase-9, and cleaved poly ADP-ribose polymerase, and the elevation of X-linked inhibitor of apoptosis protein [82].

*Polygonum cuspidatum* refers to the dried root and stem of a plant from the Polygonaceae family. As one of the famous traditional Chinese medicines (TCMs), *P. cuspidatum* is usually used in the treatment of jaundice, hyperlipidemia, infections, and inflammatory conditions [83]. The neuroprotective effects of *resveratrol* are associated with SIRT1/PGC-1 signaling in mice with TBI. The administration of resveratrol after TBI significantly reduces the expression of P-p38 MAPK 86; increases the expression of SIRT1/PGC-1 signaling, including SIRT1, PGC-1, and synaptophysin (SYN); relieves edema; and increases the ability to fight cognitive impairment [84]. Moreover, *resveratrol* remarkably improves cognitive function and reduces cerebral inflammation and edema by elevating the levels of P62, P-pI3K, P-Akt, and P-mTOR, and by reducing the levels of LC3 II, Beclin-1, IL-1β, and TNF-α [85].

*Curcuma longa* refers to the dried rhizome of a plant from the Zingiberaceae family. It has been used to treat several conditions, such as inflammation, tumor, asthma, and immune dysfunction [86–88]. The neuroprotective effects of *curcumin* are associated with p38/MAPK signaling in mice with TBI. The oral administration of *curcumin* after TBI significantly reduces the expression of p38/MAPK signaling, including p-p38 and NF-κB; decreases the expression of IL-1β, IL-6, and TNF-α; and increases the ability to fight against neurologic impairment [89]. Additionally, the administration of *curcumin* after TBI reduces spatial memory decline; increases neurogenesis; and upregulates the expression of BNDF, tyrosine kinase receptor b (Trkb), PI3K, and AKT [90].

*Salvia miltiorrhiza* refers to the dried root and rhizome of a plant from the Lamiaceae family. Salvianolic acid A is extracted from *Salvia miltiorrhiza*, China, *S. miltiorrhiza* injection, which is a standardized agent, has widely been used in clinical treatment [91–93]. and the administration of *salvianolic acid A* intraperitoneally after TBI significantly diminishes brain water content, ameliorates NSS and spatial learning and memory functioning, and reduces the levels of TNF and IL-1β, thereby exerting neuroprotective effects [94]. *Tanshinone IIA* is also derived from *Salvia miltiorrhiza*, which can significantly inhibit oxidative stress and apoptosis by the reduction of p47phox, CD11, AQP4, glial fibrillary acidic protein (GFAP), malondialdehyde (MDA), catalase (CAT), IL-1β, and TNF-α, and the elevation of SOD and GSH-PX [95].

*Artemisia annua* belongs to the Asteraceae family of plants. Until now, active compounds of *A. annua* have been evaluated in TBI models. The major active compounds of *A. annua* include flavonoids, phenolics, artemisinin, coumarins, sesquiterpenoids, and triterpenoids. Gugliandolo et al. found that *artesunate* was able to promote neuronal survival after TBI through the reduction of Nf-kb, inflammation body complex, GFAP, and Iba-1, and the elevation of BDNF, GDNF, and NT-3 [96]. In addition, the neuroprotective effects of *artesunate* in a CCI rat model showed that *artesunate* ameliorated the permeability of the blood–brain barrier and reduced brain water content and neuro-inflammation [97].

### Formulae and decoctions

Taohong-siwu decoction (THSWD), an ancient TCM herbal formula consisting of six herbs (*Carthami flos* [Honghua], *Semen persicae* [Taoren], *Angelicae sinensis radix* [Danggui], *Chuanxiong rhizoma* [Chuanxiong], *Rehmanniae radix praeparata* [Shudihuang], and *Paeoniae radix alba* [Baishao]) in a 1:1:1:1:1:1 mass ratio, was first described in Wu Qian’s Yi Zong Jin Jian during the Qing dynasty. In TCM practice, it is believed that THSWD can mitigate blood stasis in case the blood becomes too sticky and generates blood clots. THSWD is not only capable of mitigating cardiovascular disease but is also effective in alleviating TBI-induced headache, dizziness, memory deterioration, and mean peak velocity of cerebral blood flow [101]. Wang et al. found that THSWD has a valid effect on TBI-induced neurologic disorder by the activation of the Wnt/β-catenin signal pathway and the elevation of BDNF and NGF levels [102].

Xuefu Zhuyu decoction (XFZYD), an ancient classical herbal formula first reported in Yi Lin Gai Cuo by the distinguished medical scientist Wang Qingren during the Qing dynasty, is mainly composed of 11 herbs (*Semen persicae* [Taoren], *Carthami flos* [Honghua], *Angelicae sinensis radix* [Danggui], *Rehmanniae radix praeparata* [Shudihuang], *Achyranthes bidentata* [Niuxi], *Paeonia lactiflora* [Chishao], *Citrus aurantium* [Zhiqiao], *Glycyrrhiza uralensis* [Gancao], *Chuanxiong rhizoma* [Chuanxiong], *Platycodon grandiflorum* [Jiegeng], and *Bupleurum chinense* [Chaihu]) in a 4:3:3:3:3:2:2:1.5:1.5:1 mass ratio. In TCM practice, it is generally believed that XFZYD can activate blood circulation and resolve stasis during the treatment of cardiovascular disease [103]. The neuroprotective effects of XFZYD in a CCI rat model demonstrated that XFZYD mitigated cognitive disorder; improved spatial learning and memory functions; and reduced the expression of Ppfia3, Rab11a, Rnf216, and Rnf6 messenger RNA after TBI [104]. Moreover, XFZYD improved neurologic function without destroying coagulation function [105].

Buyang huanwu decoction (BYHWD), a classical formula, also prescribed by Wang Qingren in Yi Lin Gai Cuo, consists of seven herbs, namely *Astragalus* (Huangqi), *Angelicae sinensis radix* (Danggui), *Paeonia lactiflora* (Chishao), *Semen* persicae (Taoren), *Carthami flos* (Honghua), *Chuanxiong rhizoma* (Chuanxiong), and *Pheretima aspergillum* (Dilong) in the mass ratio of 40:2:2:1:1:1:1. BYHWD is commonly used to treat cerebrovascular diseases in the sequela stage because of qi insufficiency and blood stasis [106]. Wang et al. [102, 107] found that treatment with BYHWD after TBI in rats improved the pathological damage in brain tissue and decreased the levels of GFAP and Tau in serum. In addition, BYHWD was shown to be able to alleviate neurologic disorders after injury by activating the Wnt/β-catenin signaling pathway and elevating the expression levels of BDNF and NGF. Of note, the effect of BYHWD against TBI was equivalent to that of Tongqiao-huoxue decoction (TQHXD).

Wuling san (WLS), a famous classical formula first mentioned in ShangHan Lun by the distinguished medical scientist Zhang Zhongjing during the Eastern Han dynasty, consists of five herbs, including *Alisma orientale* (Zexie), *Atractylodes* macrocephala (Baizhu), *Polyporus umbellatus* (Zhuling), *Poria cocos* (Fuling), and *Cinnamomum cassia Presl* (Guizhi) in the mass ratio of 4:3:3:3:2. The neurotherapeutic effect of WLS in mice with TBI correlates with diminished water content and the elevation of messenger RNA levels of PGC-1α and NRF1, thereby promoting mitochondrial biosynthesis and enhancing the supplementation of energy to the brain tissue [108]. In addition, modified WLS could exert neurotherapeutic effects by the reduction of inflammatory cytokine contents in serum, including IL-6, IL-8, and TNF, as well as by decreasing the brain water content after TBI [109].

Wendan decoction (WDD) is a famous traditional herb formula that typically contains *Pinelliae rhizoma* (banxia 6 g), *Bambusae caulis* (zhuru 6 g), *Pericarpium citri reticulatae* (chenpi 9 g), *Fructus aurantii immaturus* (zhike 6 g), *Poria cocos* (fuling 4.5 g), *Zingiberis rhizoma* (ganjiang 5 pieces), *Jujube* (dazao 1 piece), and *Radix glycyrrhizae* (gancao 3 g). It was first described in Prescriptions Worth Thousand Gold for Emergencies by the distinguished medical scientist Sun Simiao during the Tang dynasty, and it was often used in the treatment of cardiovascular disease, gastrointestinal upset, and mental disorders [110]. In an RCT of TBI, the administration of WDD could improve neurologic function following acute TBI without generating mortality in the WDD group [111]. However, additional animal experiments and clinical trials on the efficacy of WDD in the treatment of TBI are needed.

Dachengqi Decoction (DCQD) is a classical herb pairing composed of *Rhubarb (dahuang), Fructus aurantii* (zhike), *Magnolia officinalis* (houpo), and *Mirabilite* (mangxiao) in the mass ratio of 4:2:3:3. It has been commonly used in the treatment of brain diseases, such as TBI and headache, due to wind pathogens and blood stasis. In mice with TBI, the administration of DCQD after TBI might successfully restore the intestinal mucosal barrier and enhance gastrointestinal motility by reducing the levels of diamine oxidase (DAO) and D-lactic acid [112].

Xiaochengqi Decoction (XCQD), a classical Chinese medicine, was first recorded from *Shang Han Lun* in approximately 200 AD and consists of *Rhubarb* (Dahuang), *Fructus Aurantii Immaturus* (Zhishi), and *Cortex Magnoliae Officinalis* (Houpo) in the mass ratio of 4:3:2. It is often used in the treatment of deficiency of qi and blood caused by the presence of long sores with heavy pus or blood loss. In a rat model of TBI, Peng et al. found that the administration of XCQD after injury had protective effects against brain water content and inflammatory response by reducing the levels of AQP4, and decreasing the levels of proinflammatory cytokines, including IL-1β and TNF-α [113]. The mechanism was confirmed in the subsequent study that demonstrated that XCQD had protective effects against TBI by reducing the number of harmful bacteria such as Bacteroidetes, Proteobacteria, Elusimicrobia, and Actinobacteria, and increasing the number of beneficial bacteria including Firmicutes and Spirochetes [114].

Tongqiao-huoxue decoction (TQHXD), a famous classical formula first recorded in Yi Lin Gai Cuo, consists of eight herbs (*Moschus berezovskii* [Shexiang], *Carthami flos* [Honghua], *Zingiber officinale* [Shengjiang], *Semen persicae* [Taoren], *Paeonia lactiflora* [Chishao], *Ziziphus jujube* [Dazao], *Chuanxiong rhizoma* [Chuanxiong], and *scallion* [Cong]) in a 0.15:9:9:6:6:6:6:3 mass ratio. Yi Lin Gai Cuo recorded that TQHXD can promote blood circulation and dredge the channels, especially in the treatment of blood stagnation of face and head as well as their meridians [115]. Meanwhile, Wu et al. [116] found that TQHXD had neuroprotective effects against brain injury by increase expression of CD34, p-focal adhesion kinase (FAK) and p-Paxillin proteins and elevate the angiogenesis via the VEGF pathway, which indicated the potential of TQHXD as a drug for the management of TBI. Therefore, the experimental application of TQHXD in the treatment of TBI needs to be further carried out.

### Combination treatment of physical methods with drugs

Acupuncture, a relatively well-acknowledged traditional physical therapy of TCM, has been utilized in China to relieve pain and boost body energy. Electroacupuncture (EA), which originated from traditional acupuncture in the 1930s, has been proven to significantly enhance traditional acupuncture's therapeutic effects in a range of diseases. A growing number of studies have shown that EA can help improve neurologic function after TBI by stimulating specific acupuncture points [117]. The stimulation of LI4 and LI11 acupoints on days 1, 2, 3, 4, and 5 after TBI restores sensorimotor, motor, learning, and memory deficits; recovers the levels of HDAC1 and HDAC3; and inhibits BDNF-related Akt/GSK-3 signaling in the cortex of TBI mice [118]. Liu et al. [119] found that auricular electroacupuncture combined with body acupuncture(PC6, GV26 and SP6) could significantly inhibit severe TBI-induced disorders of consciousness. Moreover, In a multicenter, single-blinded study, 250 patients with severe TBI-induced acute gastrointestinal injury received EA intervention at Zhongwan, Tianshu, bilateral Zusanli, Shangjuxu, and Xiajuxu acupoints for 30 min twice daily for 7 days in addition to conventional treatment that included mannitol, epilepsy treatment, infection prevention, and nutritional support. Scorse of gastrointestinal failure (GIF), Sequential Organ Failure Assessment (SOFA), Acute Physiology and Chronic Health Evaluation (APACHE II), GCS, Multiple Organ Dysfunction Syndrome (MODS) scores, D-lactic acid (D-lac), lipopolysaccharide (LPS), DAO, and intra-abdominal pressure (IAP) were recorded. The authors concluded that in individuals with severe TBI exacerbated by acute gastrointestinal injury, early EA and conventional treatment could enhance clinical prognosis and gastrointestinal function [120].

## Discussion

TBI is an extremely complex disease associated with multiple interacting secondary injury processes. Many promising therapeutic candidate drugs have been identified using experimental models, but, until now, none have successfully shown clinical benefits, and no drug is currently approved by the FDA for the treatment of TBI.

In this study, we summarized chemical compounds, herbal medicines, and combination treatment of physical methods with drugs, which show neuroprotective effects and therapeutic action on TBI. Some of these drugs could enter the clinical trial stage, but most were forced to terminate or withdraw in phase III or even phase II (Table 1). An international trial published in the *Lancet* reported that corticosteroids caused a higher risk of death rate within 2 weeks in patients with significant TBI. Moreover, certain complications, such as hypertension and immunosuppression, were not well controlled after taking corticosteroids, which caused the trial to declare a failure in the treatment of TBI [12]. A study in the *Frontiers in Neuroscience* reported that a large double-blinded phase III RCT [18] that investigated progesterone failed to achieve a clinical benefit in terms of postoperative mortality and functional outcomes after TBI, leading to the termination of subsequent clinical trials. In addition, progesterone caused more frequent side effects such as phlebitis or thrombophlebitis. It has been found that no serious adverse effects occurred in a phase II clinical trial of atorvastatin in mild TBI patients [34], but the treatment was not effective in the amelioration of neurologic recovery, although it has been shown in experimental studies that statins may have neuroprotective effects [28, 29]. In addition, Giorgi et al. concluded that the administration of atorvastatin for 7 days after TBI was safe but not effective in the improvement of neurologic recovery, leading to the termination in the phase II stage with the limitations of small sample size and single severity degree of TBI [34]. Although these trials seemed to suggest some progress, they failed to enter clinical phase IV and be applied for the clinical treatment of TBI.

In a prospective randomized pilot trial, Schroeppel et al. [22] found that beta-adrenergic blockade by propranolol had a protective effect on TBI and reduced the mortality rate, but there was no evidence to confirm that propranolol can be applied to the secondary injury, including intracranial hemorrhage, electrolyte disturbance, and pulmonary edema. A study published in the *Lancet* reported that TXA can reduce TBI-related deaths in patients with mild TBI who had intracranial hemorrhage on computed tomography scans within 3 h [44]. However, the study only targeted intracranial hemorrhage but not other targets, such as intracranial infection, pneumonia, pulmonary edema, and electrolyte disturbance. Presumably, the complex pathological process of TBI is the reason why the trials reported in this review failed to enter clinical phase IV and be applied for clinical treatment. Therefore, “single-compound, single-disease” drugs fail to alleviate TBI [18, 19, 43, 53].

The safety of herbal medicines for TBI should be taken into consideration. Yang et al. [77] concluded that feeding with a dose of 12 g/kg crude *Rhubarb* for 24 h alleviated the neurologic dysfunction of TBI. Furthermore, Zhang et al. [121] found that crude *Rhubarb* intragastric administration in a dose of 76 g/kg for 14 days was toxic to the hepatic and renal function of normal male juvenile mice. It is interesting to note that *Rhubarb* 's nephrotoxicity is largely related to its chemical components. The primary chemical components of *Rhubarb* were in the following sequences in terms of nephrotoxicity: *chrysophanol* > *aloe-emodin* > *physcion* > *rhein* > *emodin*. The nephrotoxicity of steamed-processed *Rhubarb* was evidently lessened. Reductions in the amount of bound anthraquinones were related to this. It indicates that for the same herb, using different extraction methods, too long administration time, and excessive dose could cause drug toxicity. Therefore, there is an urgent need to harmonize the method of herbal extraction, proper duration, and dosage to control the safety of herbal medicine.

Moreover, the described animal studies of TBI are somewhat different from clinical studies (Table 1). In the majority of the reports in this review, the main research objects of animal studies were rodents, and there are huge differences between humans and rodents in terms of anatomical morphology and metabolic characteristics. Meanwhile, the causes of TBI mainly include road injuries and falls [1–4], while the animal models relied on CCI, FPI, and WDI, which are not consistent with clinical TBI [77, 81, 98]. Furthermore, the sample size of animal studies (≧15) [46, 49] is far lower than that of clinical studies (≧57) [58], and the latter are mostly confined to the male sex; therefore, the conclusions drawn from such studies are limited. At last, their observation time and therapeutic window vary, so they cannot specifically reflect the changes in the disease course in the actual clinical treatment process.

Herbal medicine has been used for thousands of years in the treatment of neurologic disorders by focusing on a “multiple compounds, yet single target and/or disease” approach [102–116]. However, modern herbal research on TBI still has limitations. The quality standards and herbal medicine need national uniformity. In the numerous studies mentioned in this review, the herbs-producing regions and their compounds were not described in the openly published papers [74, 77, 99, 100]. Even when a source was mentioned, the catalog number was not marked, which directly represents the formulation and grade of the herbal medicine [67, 70, 75]. The source and catalog number of XFZYD were not mentioned either [104, 105]. Yang et al. [77] specified that *Rhubarb* was from Gansu Province, China, while the catalog number was not provided. Therefore, there is a need to uniform the quality standards of herbal medicine.

The subjectivity of acupuncture and EA should be strongly taken into account. Traditional acupuncture has many restrictions due to its subjectivity; for instance, therapists performing acupuncture must assure that the patient has attained a special feeling known as “de qi,” which demonstrates its curative effect. However, the therapist must change the needle's angle and depth to obtain “de qi,” which is based on the individual's experiences and is subjective. Acupuncture's effectiveness can also be difficult to measure and duplicate. Huang et al. [118] and Wu et al. [119] confirmed acupuncture’s curative effect on TBI, but none of them provided the needle's angle and depth. EA, which is characterized by parameter objectification, has therefore drawn increasing attention. However, looking for the best parameter setting is complicated. The EA therapy depends on the settings being chosen and applied correctly. Hung et al. [118] and Xing et al. [120] did not provide parameter settings of the bio-signaling of bodily responses brought on by EA activation, which could objectively monitor some physiological or biochemical changes in response to EA stimulation and observe the degree of “de qi.” Therefore, it is necessary to establish a bio-signaling feedback monitoring system to monitor the body changes and observe the degree of “de qi.”

## Conclusion

Despite these limitations, overall research is very promising and shows that herbal medicine may be potentially neuroprotective in TBI. However, it is important to provide an accurate and simple method for herbal quality control. Moreover, detailed, rigorous, and systematized preclinical evaluation should be established before successful clinical transformation of those treatment strategies. Due to the complexity of the chemical constituents of herbal medicine, it is difficult to separate them on column within a short time, so there is a challenging problem in the clinical use of on column chromatography herbal medicine injection because its constituent elements are complex. Though the use of traditional herbal medicines in the clinical setting has a long history, and their efficacy has been proven in clinical trials, it is necessary to provide an accurate and simple method for herbal quality control. Furthermore, in consideration of the therapeutic effects of EA and some drugs on TBI, the combination of acupuncture with drugs can be vigorously developed in the future.

## Data Availability

The authors declare that all data supporting the findings of this study are available within the article and its uploaded attach files.

## Abbreviations

TBI: Traumatic brain injury
FDA: Food and Drug Administration
EMA: European Medicine Agency
EPCs: Endothelial progenitor cells
GR: Glucocorticoid receptor
MR: Mineralocorticoid receptor
CRASH: Corticosteroid Randomization After Significant Head Injury
GCS: Glasgow coma scale
EPSCs: Excitatory postsynaptic currents
IPSCs: Inhibitory postsynaptic currents
ICP: Intracranial pressure
EAAT-3: Excitatory amino acid transporter 3
SOCS-3: Suppressor of cytokine signaling-3
p-STAT-3: Phosphorylated signal transducer and activator of transcription-3
NGF: Nerve growth factor
NSE: Neuron-specific enolase
COBRIT: Citicoline Brain Injury Treatment
HMG-CoA: 3-Hydroxy-3-methyl glutaryl coenzyme A
NOS: Nitric oxide synthase
TBK1: TANK binding kinase 1
TLR4: Toll-like receptor 4
GRP78: Glucose-regulated protein 78
TXA: Tranexamic acid
MIP-1α: Macrophage inflammatory protein 1α
MCP-1: Monocyte chemoattractant protein-1
CRASH-2: Clinical Randomization of an Anti-fibrinolytic in Significant Hemorrhage 2
EPO: Erythropoietin
MAPK: Mitogen-activated protein kinase
cAMP: Cyclic adenosine monophosphate
AQP4: Aquaporin 4
OFC: Orbitofrontal cortex
mPFC: Medial prefrontal cortex
NAc: Nucleus accumbens
Drd1: Dopamine receptor D1
Drd2: Dopamine receptor D2
Dnmt3a: DNA methyltransferase 3A
CCI: Controlled cortical impact
WDI: Weight drop injury
FPI: Fluid percussion injury
*P. ginseng*: *Panax ginseng*
*C. tinctorius* L.: *Carthamus tinctorius* L
*P. notoginseng* L.: *Panax notoginseng*
*PNS*: *P. notoginseng* Saponins
t-PA: Thromboplastin time
PT: Prothrombin time
APTT: Activated partial thromboplastin time
ET: Endothelin
P13K: Phosphatidylinositol 3-kinase
MMP-9: Matrix metalloproteinase 9
5-HT: 5-Hydroxytryptamin
5-HIAA: 5-Hydroxy indole acetic acid
GABA: γ-Aminobutyric acid
Glu: Glutamic acid
Asp: Aspartic acid
LDH: Lactate dehydrogenase
GSDMD: Gasdermin D
NLRP3: NOD-like receptor protein 3
ICAM-1: Intercellular cell adhesion molecule-1
TCMs: Traditional Chinese medicines
TCM: Traditional Chinese medicine
SYN: Synaptophysin
Trkb: Tyrosine kinase receptor b
GFAP: Glial fibrillary acidic protein
MDA: Malondialdehyde
CAT: Catalase
*A. annua*: *Artemisia annua*
THSWD: Taohong-siwu decoction
XFZYD: Xuefu Zhuyu decoction
BYHWD: Buyang huanwu decoction
WLS: Wuling san
WDD: Wendan decoction
DCQD: Dachengqi Decoction
DAO: Diamine oxidase
XCQD: Xiaochengqi Decoction
TQHXD: Tongqiao-huoxue decoction
FAK: Focal adhesion kinase
EA: Electroacupuncture
GIF: Gastrointestinal failure
SOFA: Sequential Organ Failure Assessment
APACHE II: Acute Physiology and Chronic Health Evaluation
MODS: Multiple Organ Dysfunction Syndrome
D-lac: D-lactic acid
LPS: Lipopolysaccharide
IAP: Intra-abdominal pressure
